# Primary Healthcare Professionals Experience of Transfer and Meaning According to Screening for Dysphagia

**DOI:** 10.3390/geriatrics4040054

**Published:** 2019-09-27

**Authors:** Alexia Andersen Fortes, Jeff André-Brylle, Signe Westmark, Dorte Melgaard

**Affiliations:** 1 Nursing home Sjælsø, 3460 Rudersdal, Denmark; jefa@rudersdal.dk; 2 Centre for Clinical Research, North Denmark Regional Hospital, 9800 Hjoerring, Denmark; s.westmark@rn.dk (S.W.); dmk@rn.dk (D.M.)

**Keywords:** nursing home, MEOF-II, workshop, elderly, transfer, meaning, dysphagia, swallowing disorders, screening

## Abstract

Transfer is a well-known theory about learning in practice contexts. This concept, combined with the need to implement screening for dysphagia in the nursing homes, has led to this project describing the experienced transfer effect and meaning among healthcare professionals after participation in a practice-orientated workshop focusing on implementing the Minimal Eating Observation Form-II (MEOF-II). Fifty-eight healthcare professionals participated in a 2.5-h facilitated practice-orientated workshop in the period from March to September, 2018. Before and after the workshop, they filled out a questionnaire that focused on the healthcare professional’s experience of skills related to dysphagia. The study documented that, after the workshop, more healthcare professionals felt competent to perform the MEOF-II to identify signs of dysphagia and know their role in screening for dysphagia. Nine months after the workshop, 80% of the residents in the nursing home had been screened for dysphagia by using the MEOF-II. This study documented that practice-orientated workshops and systematic follow-up encouraged the healthcare professionals to use the MEOF-II to contribute to the early detection of dysphagia in the nursing home. Workshops based on the transfer theory may also be relevant for implementation and application of other new skills in similar settings.

## 1. Introduction

The prevalence of oropharyngeal dysphagia (OD) is 30–40% in independently living elderly people and 44–50% in patients in acute geriatric units [1,2,3,4]. In patients with dementia, the prevalence is up to 84% [5,6]. OD may lead to aspiration pneumonia, malnutrition and dehydration [7]. The loss of function and decreased quality of life are well-known complications [8]. Older adults are particularly vulnerable to OD because the disease prevalence increases with age, and age-related changes of the aerodigestive tract affect the ability to swallow efficiently and safely [9]. Patients with OD are significantly costlier for both hospitals and municipalities compared to patients without OD [10,11]

In nursing homes in Denmark, there is a growing focus on implementing a structured and systematic screening strategy to identify problems related to OD to optimise the citizens’ quality of life, level of function, and reduce costs. One of the recommended screening tools is the Minimal Eating Observation Form-II (MEOF-II), which is a screening instrument for a structured observation of an eating situation [12]. MEOF-II is used for screening for dysphagia [12]. The Danish version of MEOF-II has been tested for reliability and validity and is psychometrically well-described in acute geriatric patients, and it includes observations related to the following categories: ingestion, deglutition and energy/appetite [13,14]. There is limited knowledge about how to improve guidelines and instil new skills in nursing homes, but when facilitating the implementation of a new practice of screening for OD, a targeted and structured approach is required [15]. Transfer is a process in which theory finds practical application in the workplace. Three factors that influence transfer are: (1) personal factors in relation to the learner, (2) factors related to the work environment and the context in which the new knowledge is to be applied and (3) factors relating to teaching and the intervention design [16,17,18].

**Figure 1:**
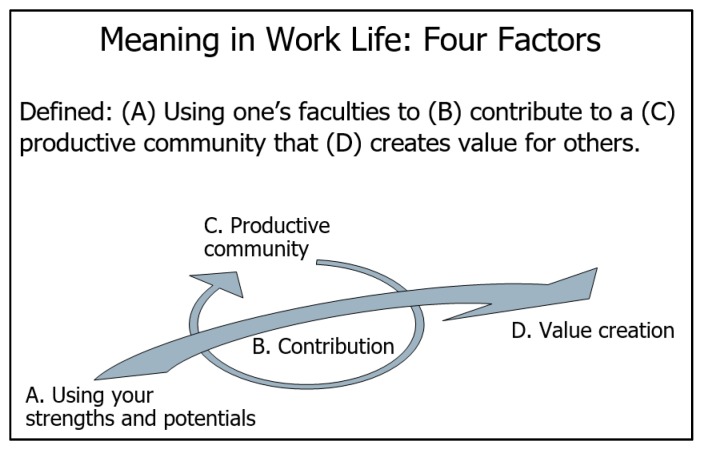
Meaning in Work Life: Four Factors [20,21]. A: Every person has a unique set of potential and signature strengths, and when actualising their potential, the meaning is produced. B: When making your contribution using the potential that is relevant to others, you can make a difference in the world. This difference produces meaning. C: Participating in a productive community or being a part of a group that accomplishes something important is experienced as meaningful. D: The identification of a need and helping to meet that need creates value for others, which, in turn, produces meaning [20,21,22,23,24].

An in-depth understanding of the material being taught combined with a clear commitment and credibility are required to promote transfer. The belief in one’s skills is an important factor in transfer as it increases the confidence and desire to apply the skills in practice [16]. Furthermore, it is an advantage that learning and the practical application take place in a social setting with others from the same or similar work areas [16]. It is essential for transfer that the person finds the new skills meaningful. This means that they identify ways in which their working practice may be improved with their application of the new skill [17]. Roy Baumeister et al. suggest four basic factors —purpose, value, efficacy and self-worth—that will result in the person experiencing that their life has sufficient meaning [19]. The definition in Figure 1 and conceptualisation of ‘the meaning in work life’ has been used:

This study aimed to describe how healthcare professionals experienced the effect of transfer and meaning after participating in a 2.5-h practice-orientated workshop focusing on OD and implementation of the dysphagia screening tool Minimal Eating Observation Form-II (MEOF-II)

## 2. Materials and Methods

### 2.1. Design

An evaluation in 2017, in the municipality of Rudersdal, showed that the effort in the municipality to implement MEOF-II as a screening tool had not been successful. No screening for dysphagia had been carried out in the municipal nursing home in which this study takes place (Nursing home Sjælsø). It was decided to change the procedure, and from March, 2018 to September, 2018, three workshops were conducted at the nursing home Sjælsø, that consists of 137 apartments and approximately 200 employees. In all, 58 healthcare professionals participated in the workshops representing nurses, social and healthcare assistants, helpers, kitchen staff, nutrition specialists and managers. Three participants (manager and kitchen staff) were not expected to screen for dysphagia after the workshop. All participants were permanent employees and worked day or evening shifts. They were selected with no consideration of their educational background. The workshops were designed and facilitated by two experienced occupational therapists (OTs) specialised in dysphagia in collaboration with the managers of the nursing home. The healthcare professionals reported their experience of transfer and meaning with the new OD screening procedures in a questionnaire consisting of eight questions (Table 1). The questionnaires were neither standardised nor validated.

The overall intervention consisted of three phases.

(1) Planning and designing the overall intervention and workshop.

This phase included:

- Defining the purpose.
- Introduction to a structured strategy to support early detection of OD,
- Introduction to MEOF-II and documentation of the findings,
- Definition of interdisciplinary responsibilities in relation to OD,
- An illustration of the chronological procedures for early detection of OD in the nursing home.

(2) Experienced OTs facilitated the structured and chronologic practice-oriented workshop to an interdisciplinary group of a maximum of 24 healthcare professionals.

Workshop schedule:

- Basic theory about OD (10–15 min)
- Sitting position (20 min)
- Interdisciplinary intervention—procedures and organisation in the municipality: A staff member from the kitchen presented modified food, and OTs presented modified drinks as well as specialised eating and drinking utensils (40 min)
- Introduction to MEOF-II (25 min)
- Evaluation and questions (15 min)

After each section of the workshop, time was allocated for discussion and reflection about the experience and its significance and use in practice.

(3) Follow-up, evaluation and assistance related to specific cases or the general use of MEOF-II were offered as needed as well as at the weekly interdisciplinary meetings.

### 2.2. Data Collection

All participants filled out a questionnaire at the beginning of the workshop consisting of the questions listed in Table 1. Three to four weeks after participating in the workshop, the participants filled out a second questionnaire containing similar questions. The second questionnaire was handed out and collected by the OTs during the weekly interdisciplinary meetings. The questionnaires were developed by the same OTs who designed and facilitated the workshop and follow-up.

### 2.3. Data Analysis

The data analysis was based on descriptive analysis of the questionnaires. The percentages for answers in each question were calculated, and the answers from before the workshop were compared with the answers from after the workshop. Answers from before and after the workshop are reported as one group, respectively, due to the size of the groups of different professions. Data is not collected as paired data and, therefore, data is reported as a difference in percentages from before and after the workshop. The results are reported as the sum of answers 4 and 5 in the questionnaire (Table 1). No statistical analyses have been made due to the number of participants.

### 2.4. Research Ethics

The participants in the project were informed verbally about the project, and according to Danish legislation, there is no requirement for consent to a survey where no person-related data is included. The surveys were stored and will be destroyed in accordance with the General Data Protection Regulation.

## 3. Results

Table 2 shows the number of participants represented in the different professions. The groups are relatively small. Therefore, we do not report results from each group of participants.

The results from before and after the workshops are documented in Table 3. The results are the sum of options four and five (see Table 1).

Nine months after the workshop, 106 out of 132 (80%) of the residents had been screened for OD with the MEOF-II.

Seven participants required extra assistance to use MEOF-II. The seven were all non-native speakers of Danish, and the assistance consisted of explaining phrases or words in relation to the MEOF-II screening tool.

## 4. Discussion

This study reported a positive transfer effect and meaning experienced among healthcare professionals after participation in a 2.5-h practice-orientated workshop with follow-up. The participants’ experiences were reported by a non-validated questionnaire which is the main limitation of this study [25]. The questionnaire was developed by consciously defining and considering the purpose, the target group, the length and simplicity of the questions and always providing an option to give a neutral answer [26,27]. Another limitation is that the data collected is not paired, which means that it is possible to see the changes only at a group level. Words such as transfer and meaning were not used to avoid subjective understandings.

The response rates are high from this broad group of professions. All participants fulfilled the questionnaire when they showed up for the workshop. The lower response rate for the follow-up questionnaire may be explained by some of the participants having taken another job, facing language difficulties, having forgotten to answer the questionnaire, or not seeing the meaning of answering it.

Before the workshop, 17% of the participants reported they felt equipped to use the MEOF-II; after the workshop, 94% answered they felt better equipped to use the MEOF-II (question 6). This success rate may relate to the promotion of transfer through the participants’ practicing the use of MEOF-II during the workshop, the possibility of the OTs offering follow-up and assistance in the subsequent weeks after the workshop, and lastly, the fact that learning and application took place in a close group of co-workers with the same or similar work-related responsibilities. [16,28,29,30].

The results show a high increase of participants experiencing more awareness of their role as well as that of their colleagues in the early detection of OD (questions 1 and 2). Uncertainty of one’s role or the lack of knowledge about the early detection of OD can hinder action, despite the obvious value, thus lowering transfer and meaningfulness [16,18,21].

According to the ‘feeling capable of observing signs of dysphagia’ and ‘feeling capable of acting accordingly when observing signs of dysphagia’ (questions 7 and 8), this study documented an increase in feeling more capable.

Nine months after the workshop, 80% of the residents in the nursing home had been screened. There is no information about the residents not screened, the quality of the screening, whether MEOF-II reduced the incidence of pneumonia, or about the impact of malnutrition and dehydration.

## 5. Conclusions

This study demonstrates that the participants experienced good transfer as a result of participation in the facilitated 2.5-h practice-orientated workshop focusing on dysphagia with follow up among healthcare professionals in a nursing home. The present study also demonstrates that the facilitated workshops and systematic follow-up supported the implementation of MEOF-II and that transfer succeeded as data shows that after nine months 80% of the residents had been screened.

The participants became more aware of their role as well as the value of their contribution, and the management secured and prioritised the resources to allow the participants to make their contribution in a productive interdisciplinary community.

The structured and well-planned workshop based on the theory of transfer has shown good results in a nursing home setting, but there is still a need for more research to ensure the sustainability when implementing new interdisciplinary procedures in nursing homes.

## Figures and Tables

**Table 1:** Questionnaire handed out before and 3-4 weeks after the workshops.

Question Number	Questionnaire before Workshop	Questionnaire 3 Weeks after the Workshop	Possible Answers
1	How clearly do you see what your role is in working with early detection of dysphagia?	How clearly do you see what your role is in working with early detection of dysphagia?	(5) Very clearly (4) Fairly clearly (3) Somewhat clearly (2) Not particularly clear (1) Not clear at all
2	How clearly do you see what role your colleagues have in working with early detection of dysphagia?	How clearly do you see what role your colleagues have in working with early detection of dysphagia?	
3	To what extent do you see your role as being important in working with early detection of dysphagia?	To what extent do you see your role as being important in working with early detection of dysphagia?	(5) Very high extent (4) Fairly high extent (3) Neither high nor low extent (2) Low extent (1) Very low extent
4	To what extent do you see your colleague’s role as being important in working with early detection of dysphagia?	To what extent do you see your colleague’s role as being important in working with early detection of dysphagia?	
5	To what extent do you believe that the early detection of dysphagia can create value for the residents.	To what extent do you believe that the early detection of dysphagia can create value for the residents.	
6	To what extent do you agree with the following statement: ‘I feel adequately equipped to use the screening-tool MEOF-II’.	To what extent do you agree with the following statement: ‘After participating in the workshop, I feel adequately equipped to use the screening-tool MEOF-II’.	(5) Highly agree (4) Mostly agree (3) Neither agree nor disagree (2) Disagree (1) Highly disagree
7	To what extent do you agree with the following statement: ‘I feel capable of observing signs of dysphagia’.	To what extent do you agree with the following statement: ‘After participating in the workshop, I feel more capable of observing signs of dysphagia’.	
8	To what extent do you agree with the following statement: ‘I feel that I am capable of acting accordingly when I observe signs of dysphagia’.	To what extent do you agree with the following statement: ‘After participating in the workshop, I feel that I am more capable of acting accordingly when I observe signs of dysphagia’.	

**Table 2:** Overview of the distribution of professions answering the questionnaire before and after the workshop.

Profession	Questionnaire 1	Questionnaire 2
	*N* = 58	*N* = 48
Nurse	8	8
Social and healthcare assistant	9	9
Social and healthcare helper	23	17
Unskilled *	6	6
Miscellaneous **	12	8

* Unskilled group consists of co-workers who do not have an education as a healthcare giver. They assist the healthcare giver. ** Miscellaneous group consists of managers, students, nutrition specialists, and kitchen staff.

**Table 3:** All participants in the workshops answered a questionnaire before and after the workshop. *N* is the sum of total answers of the questionnaire, and *n* (%) is the response rate and is the sum of options 4 and 5 in the questionnaire.

Question	Before Workshop	After Workshop
	*N* = 58, *n* (%)	*N* = 48, *n* (%)
1	19 (33%)	37 (77%)
2	27 (47%)	36 (75%)
3	49 (85%)	46 (96%)
4	49 (85%)	45 (94%)
5	49 (85%)	46 (96%)
6	10 (17%)	45 (94%)
7	33 (57%)	47 (98%)
8	39 (67%)	45 (94%)
